# Berberine Mediates Exosomes Regulating the Lipid Metabolism Pathways to Promote Apoptosis of RA-FLS Cells

**DOI:** 10.3390/ph17111509

**Published:** 2024-11-09

**Authors:** Si-Fan Guo, Zhi-Bo Wang, Dan-Dan Xie, Ying Cai, Yan Wang, Xian Wang, Qiang Yang, Ai-Hua Zhang, Shi Qiu

**Affiliations:** 1 International Advanced Functional Omics Platform, Scientific Experiment Center, Hainan Medical University, Haikou 570100, China or hy0303019@muhn.edu.cn (S.-F.G.); wangzhibo0720@163.com (Z.-B.W.); hyyfy2316@muhn.edu.cn (D.-D.X.); hy0303015@hainmc.edu.cn (Y.W.); 15500943749@163.com (X.W.); 2 GAP Center and Graduate School, Heilongjiang University of Chinese Medicine, Harbin 150040, China; hljzyyyangqiang@126.com

**Keywords:** berberine, exosomes, rheumatoid arthritis, mechanism of action

## Abstract

**Objectives**: Rheumatoid arthritis (RA) is a chronic autoimmune disease characterized by joint damage and commonly linked to symptoms such as inflammation, swelling, and pain. Traditional Chinese Medicine offers complementary and integrative approaches in the management of rheumatoid arthritis, potentially providing additional options that may help address treatment challenges and enhance overall patient care. This paper explores the mechanism of action of berberine from the perspective of cellular exosomes by mediating exosomal contents and thus treating RA. **Methods**: With the help of flow cytometry and confocal laser scanning microscope, it was determined that berberine promotes apoptosis in RA-FLS cells, and then lipid metabolomics technology was applied to screen and characterize the exosomes of RA-FLS cells to identify lipid core biomarkers closely related to RA, which were then projected into various databases for comprehensive analysis. **Results**: The data analysis showed that berberine could call back 11 lipid core biomarkers closely associated with RA, and interactive visualization of the database revealed that these markers were mainly focused on lipid metabolism aspects such as fatty acid elongation, degradation, and biosynthesis, as well as the biosynthesis of unsaturated fatty acids or PPARA activation of gene expression, PPARα‘s role in lipid metabolism regulation, glycerophospholipid metabolism, mitochondrial fatty acid oxidation disorders, and organelle biogenesis and maintenance. **Conclusions**: Berberine exerts its therapeutic effect on RA by mediating exosomal contents and thus regulating multiple lipid-related biological pathways, affecting the PPARγ-NF-κB complex binding rate, CREB and EGR-1 expression, cellular phagocytosis, and other aspects needed to inhibit proliferation and inflammatory responses in RA-FLS. This study offers a research foundation for exploring the mechanism of action of berberine in the treatment of RA.

## 1. Introduction

RA is a chronic autoimmune illness that primarily involves the joints and presents as chronic symmetrical polyarticular inflammation [1]. While systemic immune dysregulation is the underlying cause of RA, the condition is primarily characterized by persistent inflammation of the synovium and subsequent joint damage, resulting in joint deformities and functional impairment [2,3]. Abnormal inflammatory activation found in the synovium of RA individuals leads to a proliferation of synovial fibroblast-like synoviocytes (FLSs), and the persistence of FLS cells in RA makes joint inflammation difficult to control [4]. FLS cells secrete a variety of cytokines and matrix metalloproteinases that promote local inflammatory and immune responses while causing damage to articular cartilage and bone tissue, promoting joint destruction and loss of function. FLS cells are able to interact with T and B cells to enhance the autoimmune response and exacerbate inflammation. Exosomes in synovial fluid from RA patients have been found to be useful as diagnostic markers for RA [5,6]. FLS cell-derived exosomes exacerbate RA development by enhancing N6-methyladenosine modification of SOX9 in chondrocytes and macrophage glycolysis [7].

Exosomes, as a type of extracellular vesicle (EV), play a crucial role in the transfer of biologically active molecules such as lipids, proteins, metabolites, and various types of ribonucleic acid, including mRNA, miRNA, and lncRNA [8]. Exosome contents can be secreted and taken up by most cell types as paracrine signals to control intercellular communication. Through the process of exocytosis, exosomes transport molecules originating from parental cells through the bloodstream and lymphatics to target cells or are released into the extracellular space, which in turn affects the metabolism and phenotype of the target cells or reveals the biological condition of the parental cells [9,10,11]. This aspect is crucial for maintaining homeostasis and facilitating communication between cells and tissues in both in vivo and in vitro settings, particularly in the context of disease. The cellular microenvironment affects the biosynthetic components of exosomes and their secretion. Exosomes have the ability to preserve information from their parent cells, which makes them potentially valuable as disease biomarkers [12,13,14]. It has been demonstrated that exosomes are crucial to the physiological and pathophysiological mechanisms of RA. For example, TNF-α-induced exosomes from FLS cells can promote HUVEC migration, invasion, and angiogenesis by targeting the miR-200a-3p/KLF6/VEGFA axis [15]. The exocrine results of FLSs stimulated by IL-1 showed that the inflammatory results changed [16]. In RA scenarios, exosomes have antigenic actions such as inducing death, delivering antigens to T lymphocytes, and regulating extrinsic damage [10]. Owing to recent advancements in high-throughput capabilities technologies, it is now conceivable to examine the metabolic composition of exosomes.

Abnormal adaptive immunity is considered to be the driving force for RA to develop from preclinical disease to obvious synovitis, in which FLS cells, the most significant matrix component, have attracted attention in the pathogenesis of RA [2]. Understanding the relationship between exosomes and FLS cells and using synovial-derived exosomes as diagnostic biomarkers for RA to predict disease stage provide new strategies for the evaluation and management of RA.

**Figure 1:**
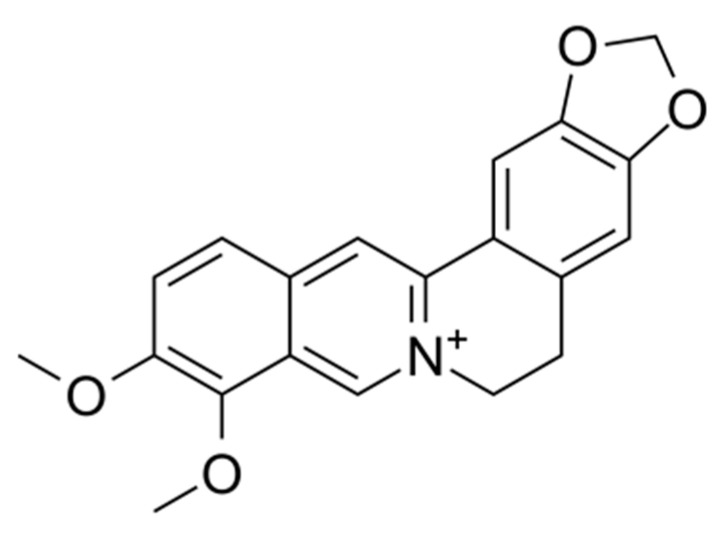
Structure of berberine.

RA is usually treated clinically with immunosuppressive drug monotherapy or combinations such as non-steroidal anti-inflammatory drugs (NSAIDs), glucocorticoids, disease-modifying anti-rheumatic drugs (DMARDs), and newer biologics [17]. Although these medicines have had some therapeutic success, their prolonged administration to patients is hindered by significant adverse effects, including infection, hepatic injury, gastrointestinal damage, and cardiac failure [18]. Traditional Chinese medicine has been utilized extensively as a complementary and alternative method for the management of RA. Today, many therapeutic drugs are based on the structure of plant extracts in clinical therapy. The chemical structure of berberine (BBR) is an isoquinoline alkaloid with a tetrahydroisoquinoline skeleton with a cationic nitrogen; its structure also includes hydroxyl and methoxy groups, and the presence of these functional groups gives it strong fat-soluble properties (Figure 1). Berberine is found in a diverse range of medicinal plants, such as Amur Cork-tree Bark, Rhizoma Coptidis, and berberis leaf, and it is now widely used to treat inflammation and infections [19]. The lipid-soluble nature of berberine allows it to effectively penetrate cell membranes and influence intracellular signaling. Its molecules are able to bind to a variety of intracellular targets, thus exerting biological effects that regulate inflammatory and immune responses [20]. Research has demonstrated that berberine possesses the ability to hinder the growth and attachment of FLS cells by modulating the ras/MAPK/foxo/HIF-1 communication pathway [21]. Berberine can ameliorate the IL-21/IL-21R-mediated autophagy invasion of FLS cells and regulate the unbalanced hhhjactivity of Th17/Treg in RA [22]. Berberine can alleviate ollagen-induced arthritis (CIA) by improving oxidative inflammation and joint injury in a mouse model mediated by the GSK 3 β/STAT/Akt/MAPKs/NF-κB signal axis [23]. More and more experimental data have demonstrated that berberine can slow down the development of RA by inhibiting joint inflammation and inflammatory signaling cascades through immunomodulation [24]. However, it is unknown whether berberine can treat RA by affecting exosomal effects. This study employed FLS cells to investigate the mechanism by which berberine acts on RA using a cellular metabolomics approach. The findings offer insights into the target and mechanism of berberine’s therapeutic effects in treating RA, highlighting the significance of FLS cells in RA prevention and treatment.

## 2. Results

### 2.1. Berberine Promotes Apoptosis of RA-FLS Cells

**Figure 2:**
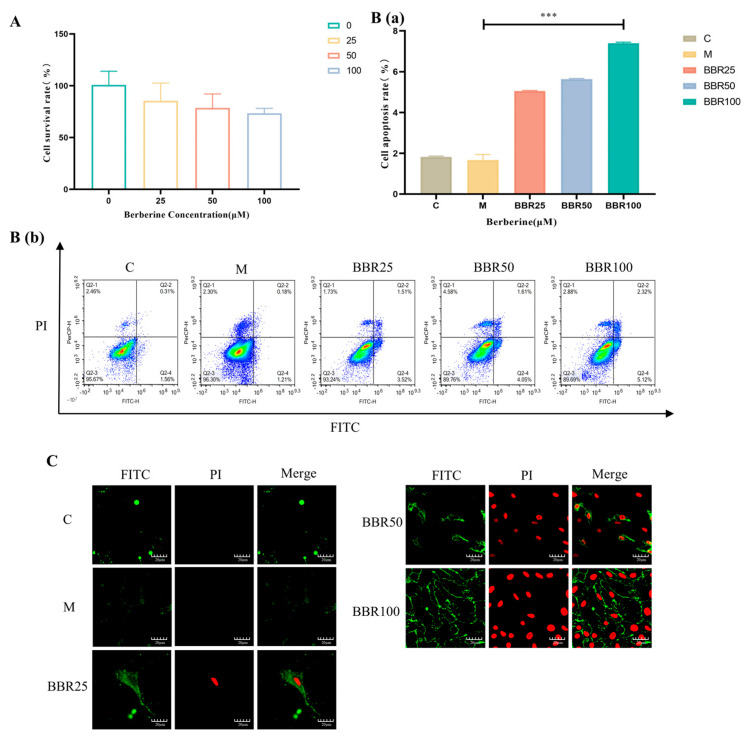
Different concentrations of berberine all promoted TNF-α-induced apoptosis of RA-FLS cells (model group, 25 μM, 50 μM, and 100 μM concentrations of berberine group) (**A**) Berberine cytotoxicity assay with CCK-8 reagent (*n* = 4 per group). (**B**) Analysis of Annexin V-FITC/PI staining in RA-FLS cells by flow cytometry (*** *p* < 0.001) (n = 3 per group; all experiments were made in triplicate). (**C**) Representative images of RA-FLS apoptosis in the berberine group at different administered concentrations in the model group as detected by the Annexin V-FITC/PI assay (scale bar represents 20 μm, PI red, Annexin V-FITC green).

To investigate the effect of berberine on the viability of RA-FLS cells, 0, 25, 50, and 100 μM of berberine was given to treat FLS cells for 24 h. The IC50 of berberine was calculated as 138.8 μM using a non-linear regression fitting curve model. Compared with the blank group, there was no significant difference in the effect of berberine at concentrations of 25, 50, and 100 μM on FLS proliferation (Figure 2A). The concentration of berberine used in this experiment was within the safe range.

To evaluate the pro-apoptotic effect of berberine in RA-FLS cells, different berberine concentrations of high, medium, and low administration groups were formulated using a basal medium containing 10 ng/mL TNF-α at 25 μM, 50 μM, and 100 μM concentrations, respectively, and incubated in an incubator for 24 h. The results are shown in Figure 2. Flow cytometry was utilized to evaluate the impact of BBR on TNF-α-induced apoptosis in RA-FLS cells. The results showed that TNF-α treatment did not induce apoptosis as opposed to cells within the blank group, whereas the 25 μM, 50 μM, and 100 μM BBR administration groups significantly promoted TNF-α-induced apoptosis in RA-FLS cells (Figure 2B) with a dose-dependent trend. Simultaneous observation of Annexin V-FITC/PI staining results revealed that the low, medium, and high doses of BBR administration groups significantly induced apoptosis in RA-FLS cells (green fluorescence for early apoptotic cells and red + green for late apoptotic cells) compared with the corresponding control cells and showed a dose-dependent trend, which was in line with the trend of the results of flow cytometry experiments (Figure 2C). These results suggest that BBR can promote apoptosis in RA-FLS cells. Considering that too high a concentration of BBR would lead to excessive cytotoxicity, 50 μM BBR was chosen as the administration concentration for subsequent experiments.

### 2.2. Differential Metabolites in FLS Cell Exosomes

**Figure 3:**
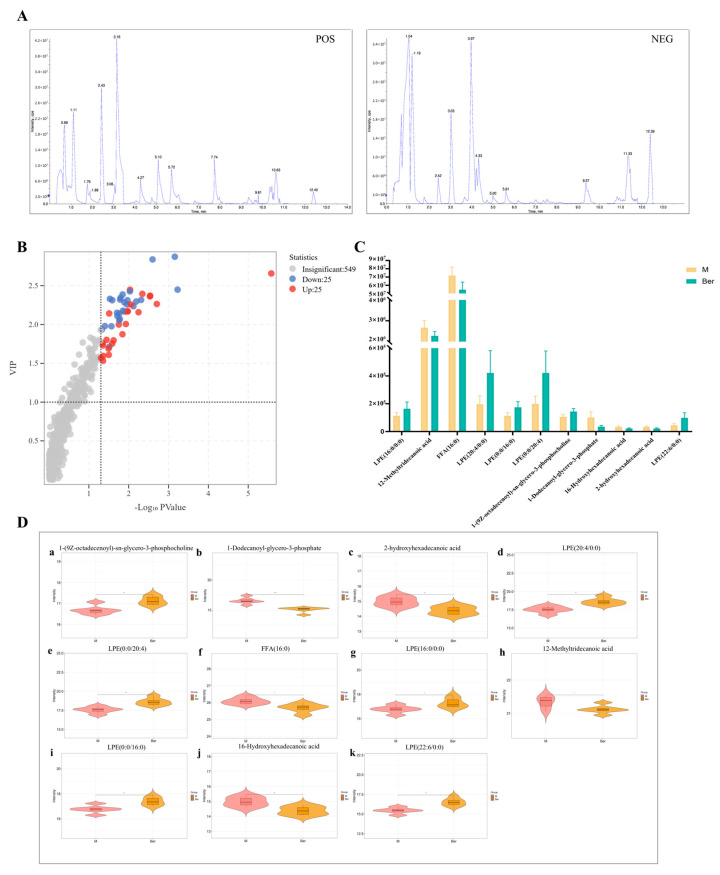
Identification of differential metabolites in extracellular matrix of the model group and the berberine group. (**A**) Base peak ion chromatograms of mixed sample QC samples in positive ion mode and negative ion mode. (**B**) Volcanic map of differential metabolites. (Each dot in the volcanic diagram represents a metabolite, where purple dots represent down-regulated differential metabolites, red dots represent up-regulated differential metabolites, and gray represents detected metabolites with no significant difference). (**C**,**D**) The relative content charts of 11 lipid biomarkers in the model group and the berberine group as well as the violin chart of the relative content of a single substance were identified.

After treating the samples according to the treatment method, the cellular exosome samples were fully scanned in positive and negative ion modes using the established analytical method. After processing the samples according to the treatment method, the cellular exosome samples were fully scanned in positive and negative ion patterns using the established analytical method to obtain a metabolic profile total ion current (TIC) map reflecting the information of the samples (Figure 3A). The three elements of statistical significance measures of *p*-value, magnitude of change, and multivariate statistical VIP values were utilized in the Metware platform (https://cloud.metware.cn) URL (accessed on 28 May 2024) statistical tests and were then combined to obtain a volcano plot that visually reflects the degree of contribution to change in the metabolic profile trajectory, thus quickly and intuitively identifying data points (metabolites, etc.) that have a large magnitude of change, are statistically significant, and have a large degree of contribution to model interpretation (Figure 3B). Among the 599 differential metabolites, 50 biomarkers were screened for significant correlation in the exosomes of the RA model group and the berberine administration group, of which 11 lipid biomarkers were identified using VIP > 1 and *p* < 0.05 as the conditions. Compared with the model group, six core differential metabolites, including LPE (16:0/0:0), LPE (0:0/20:4), LPE (20:4/0:0), LPE (0:0/16:0), LPE (22:6/0:0), and 1-(9Z-octadecenoyl)-sn-glycero-3-phosphocholine, showed an increasing trend in the berberine group. The five core differential metabolites of 12-methyltridecanoic acid, FFA (16:0), 16-hydroxyhexadecanoic acid, 2-hydroxyhexadecanoic acid, and 1-dodecanoyl-glycero-3-phosphate showed a decreasing trend. The peak intensities of each substance were plotted in Figure 3C, and the contents of the 11 substances were plotted in Figure 3D. These metabolites and their corresponding compound names, masses (*m*/*z*), and molecular formulae are summarized in Table 1. The above 11 metabolites were considered potential biomarkers for berberine to ameliorate TNF-α-induced metabolic changes in RA-FLS.

### 2.3. Metabolic Profile of FLS Cell Exosomes

**Figure 4:**
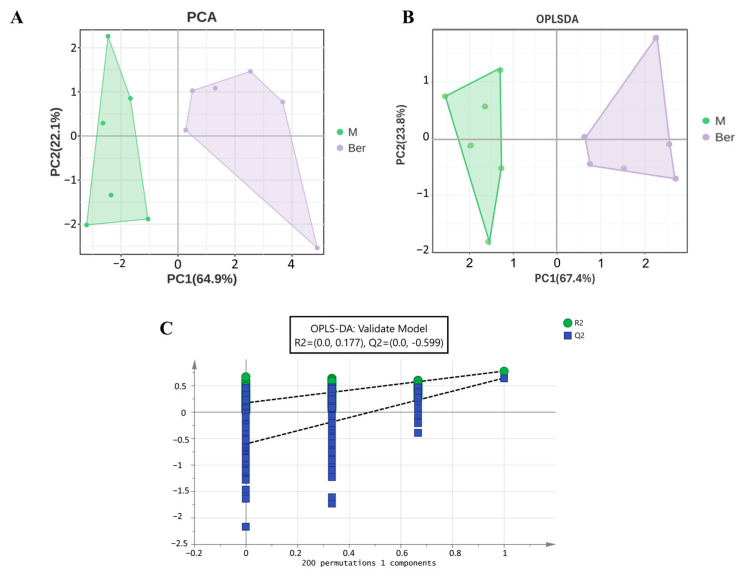
Multivariate statistical analysis of differential metabolites. (**A**) PCA, (**B**) OPLS-DA, (**C**) scatter plot and OPLS-DA substitution test plot of 11 lipid biomarkers.

Metabolic profiles can effectively reflect the overall metabolic disorder status of RA-FLS cells. Based on the 11 lipid biomarkers selected above, the peak detection and peak alignment data were normalized using the “PCA and OPLS-DA” module in the OmicShareto obtain the PCA and OPLS-DA score plots of exosomes reflecting the degree of dispersion between groups (Figure 4A,B). OPLS-DA is based on PLS-DA with orthogonal transformation correction, which can filter out the noise unrelated to the classification information. This correction has the potential to significantly reduce the model’s complexity and improve its explanatory capabilities without compromising its predictive capabilities, thereby allowing for the most comprehensive examination of the distinctions among groups. From the PCA score plot and the OPLS-DA score plot, it was possible to discover that both the model group and the berberine group could be separated and that the distribution points of samples between the groups were closer and less different, indicating that, after berberine treatment, there existed significant variation in these 11 biomarkers between the model group and berberine group. To verify the reliability of the model group, the OPLS-DA substitution test was performed. The horizontal coordinates indicate the similarity between the true grouping of the samples and the 200 random groupings; the vertical coordinates indicate the model evaluation parameters; and the Q2 and R2 points in the upper right corner indicate the model evaluation parameters for the true grouping. In Figure 4C, it can be seen that the predicted R2 and Q2 points are lower than the original R2 and Q2 points on the upper right, and the intercept of the regression line for Q2 is <0.05, indicating that the replacement test passes and the model is not overfitted.

### 2.4. Berberine-Regulated Metabolic Profile of FLS Cell Exosomes

**Figure 5:**
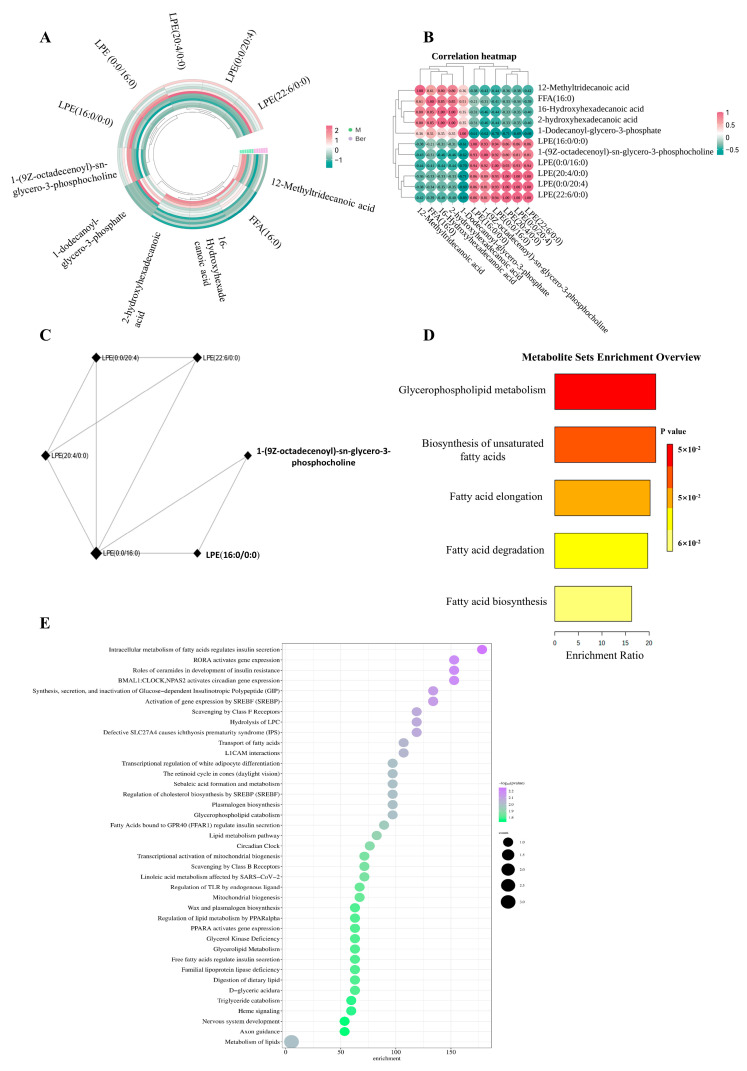
Analysis of eleven lipid biomarkers combined with online databases. (**A**) Cluster analysis of eleven lipid biomarkers in the model group and the berberine group (M: model group; BER: berberine group). (**B**) Correlation analysis of eleven lipid biomarkers in the M group and the BER group (M: model group; BER: berberine group; pink is positively correlated; green is negatively correlated). (**C**) The depolarization sparse partial correlation algorithm (DSPC) module in the MetaboAnalyst network data processing platform analyzes the correlation of core biomarkers, and the more nodes, the higher the correlation. (**D**) Eleven lipid biomarkers were enriched and analyzed based on KEGG database. The color of the histogram represents the enrichment significance *p*-value, and the abscissa represents the enrichment ratio. (**E**) Eleven lipid biomarkers were enriched and analyzed based on the RaMP-DB database. The circle color represents the enrichment importance *p*-value, whereas the circle size shows the quantity of chemicals in the RaMP-DB pathway.

The component names, mass spectrum peak intensity information, lipid classification information, and group information of 11 core lipid differential metabolites between the model group and the berberine group were respectively imported into BioDeep 1.0, Metware 1.0, and the MetaboAnalyst 5.0 network data processing platform for preprocessing, and then these data were imported into the “cluster heat map” module and the “correlation heat map” module for analysis. Based on the expression of 11 substances in different samples, hierarchical clustering analysis was carried out by using the Ward algorithm to calculate Euclidean distance, and it was found that these substances could be obviously clustered into two categoriesindicating that the samples had strong correlation and good biological repetition (Figure 5A). 

The median correlation analysis of 11 core distinctive metabolites among the model group and the berberine group showed that 11 core differential metabolites could be clustered within the group, among which LPE lipids and 1-(9Z-octadecenoyl)-sn-glycero-3-phosphocholine were negatively correlated in the model group, while the remaining five metabolites were negatively correlated in the berberine group; the results were shown by thermal graphs (Figure 5B). Eleven core lipid differential metabolites were introduced into the MetaboAnalyst 5.0 network data processing platform, and the depolarization sparse partial correlation algorithm (DSPC) module was adopted. The modular algorithm analyzes the correlation degree of core biomarkers based on the sparse lasso modeling process. The system is a combination of gene, metabolite, and disease interaction systems. Based on the analysis results, it was found that LPE lipids and 1-(9Z-octadecanoyl)-sn-glycero-3-phosphocholine were strongly correlated. Combined with the results of the correlation analysis, LPE lipids and 1-(9Z-octadecanoyl)-sn-glycero-3-phosphocholine in the model group showed a negative correlation, indicating that these metabolites were in RA (Figure 5C). 

Eleven lipid core biomarkers of exosomes were enriched and analyzed by the Enrichment Analysis module in MetaboAnalyst. Based on the KEGG database and the SMPDB (Small Molecular Pathway Database) interactive visual database, it was found that the 11 core biomarkers were mainly focused on glycerophospholipid metabolism, unsaturated fatty acid biosynthesis, and fatty acid extension, degradation, and biosynthesis, as well as phospholipid biosynthesis, fatty acid extension in mitochondria, fatty acid metabolism, steroid biosynthesis, and bile acid biosynthesis (Figure 5D). Based on the multi-source integrated RaMP-DB database (relational database of metabolic pathways), it was found that the 11 core biomarkers were mainly focused on the intracellular metabolism of fatty acids, lipid metabolism, glycerophospholipid catabolism, PPARA activation gene expression, PPARα regulation of lipid metabolism, mitochondrial fatty acid oxidation disorder, and organelle biogenesis and maintenance (Figure 5E). 

## 3. Discussion

This study discovered that berberine might dramatically suppress the proliferation of RA-FLS fibroblasts. The biological effect of berberine in RA-FLS cells was confirmed by choosing 50 μM as the experimental concentration. Eleven lipid biomarkers were screened and identified by scanning the extracellular exosomes of the model group and the berberine group in positive and negative ion mode, including LPE (16:0/0:0), LPE (0:0/20:4), LPE (20:4/0:0), LPE (0:0/16:0), LPE (22:6/0:0), 1-(9Z-octadecenoyl)-sn-glycero-3-phosphocholine, 12-methyltridecanoic acid, FFA (16:0), 16-hydroxyhexadecanoic acid, 2-hydroxyhexadecanoic acid, and 1-dodecanoyl-glycero-3-phosphate.

**Figure 6:**
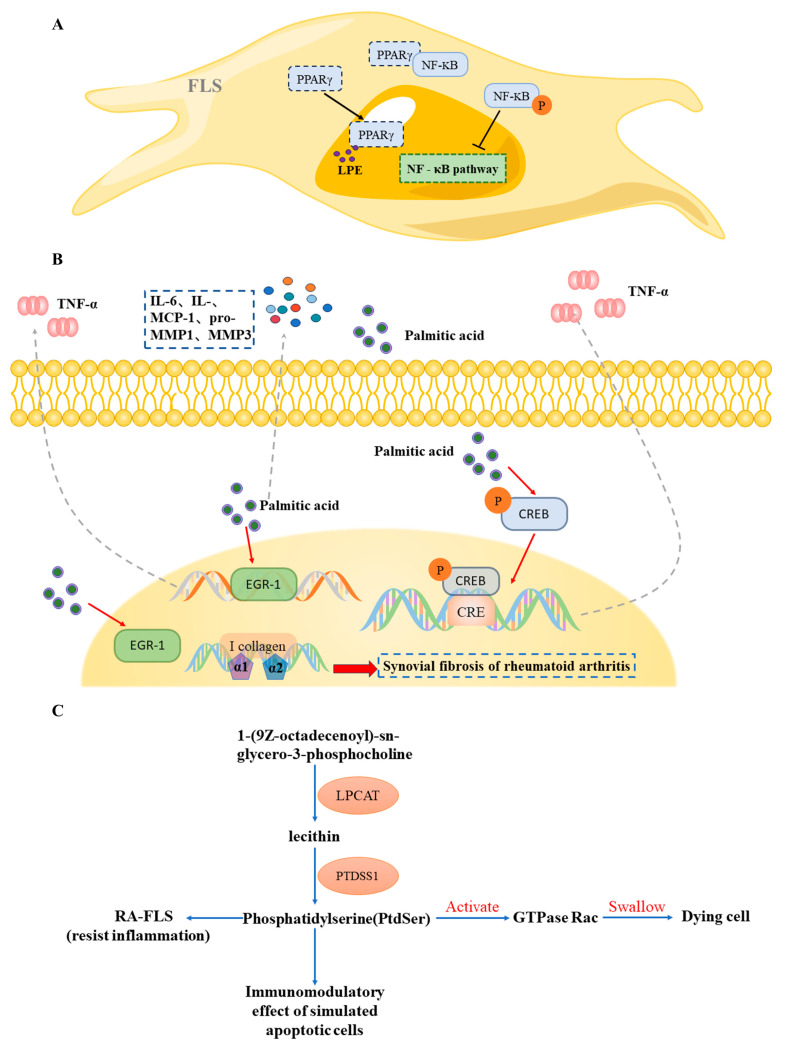
The mechanism of berberine in the therapy of RA by mediating the contents of exosomes. (**A**) LPE lipids can competitively bind to PPARγ with NF-κB. Berberine inhibits the separation of the PPARγ-NF-κB complex by up-regulating the content of LPE in exosomes, forming a protein–protein interaction and controlling the expression of the NF-κB pathway, thus affecting the proliferation of FLS. (**B**) Palmitic acid can promote the release of chemokines and cytokines that cause inflammation and stimulate the activation of CREB and EGR-1; the over-expression of EGR-1 activates the genes encoding α1 and α2 chains of type I collagen, which leads to synovial fibrosis. Berberine can affect the above process by down-regulating the content of palmitic acid. (**C**) The compound 1-(9Z-octadecenoyl)-sn-glycerol-3-phosphocholine can be transformed into PtdSer, which activates GTPase Rac and phagocytizes dying cells. Berberine can accelerate the phagocytosis of dying cells by up-regulating the content of 1-(9Z-octadecenoyl)-sn-glycerol-3-phosphocholine, thus affecting the progress of RA. (Note: (**A**) Light yellow and yellow represent cytoplasm and nucleus; blue stands for protein; green represents the signal path; purple stands for LPE substance. (**B**) Light yellow and yellow represent the cell membrane and nucleus; green, blue, and orange represent proteins; Pink represents TNF-α; Colored circles represent proinflammatory factors; Green and purple concentric circles represent palmitic acid. (**C**) Orange stands for enzyme).

As a lipid metabolite, lysophosphatidylethanolamine (LPE) is produced by various cells and participates in various immunoregulatory reactions. At the same time, from the molecular structure, LPE has hydrophilic and hydrophobic groups of phosphatidylethanolamine, and the reduction of hydrophobic groups greatly improves its hydrophilic properties, making it have good antibacterial and antioxidant capabilities [25]. The results show that LPE has a significant protective effect on LPS-induced cell inflammation and oxidative stress. In the PPARγ signaling pathway, the upstream ligand of PPARγ can be an unsaturated fatty acid, and it was found that the LPE lipid, as an unsaturated fatty acid, can compete with NF-κB to bind PPARγ and “capture” it from the cytoplasmic PPARγ-NF-κB complex [26]. In comparison with the model group, the content of free LPE in the berberine group increased, indicating that PPAR-γ is more likely to directly combine with the subunit p65/p50 of NF-κB, resulting in a protein–protein interaction, forming a transcription inhibition complex, reducing the binding activity of NF-κB to DNA, and inhibiting the expression of the NF-κB pathway [27]. PPARγ expression can be down-regulated in RA synovial tissue, inhibiting FLS growth and activation in adjuvant-induced arthritis via the Wnt/β-catenin communication pathway [28]. PPAR-γ decreases FLS activation and inflammatory variables via the p53 pathway, enhancing the immune system’s response of RA patients [29] (Figure 6A). 

The secretion of inflammatory cytokines such as IL-6, chemokine IL-8, monocyte chemoattractant protein 1, and matrix metalloproteinases pro-MMP1 and MMMP3 increased in a dose-dependent way as the synovial fibroblasts of RA received stimulation with palmitic acid and linoleic acid [30]. Stimulation of mouse osteoblasts with palmitic acid alone can significantly increase the secretion of IL-6, IL-8, and MCP-1 [31]. In comparison with the model group, the content of palmitic acid in the berberine group decreased, which further indicated that the berberine group could reduce the release of inflammatory cytokines such as IL-6, chemokine IL-8, and MCP-1 after administration. The experimental results of Thais Martins de Lima-Salgado [32] show that palmitic acid can rapidly stimulate the activation of CREB and EGR-1 after 15 min without LPS, further increase the activation induced by LPS, and further promote the production of TNF-α. As an extracellular enzyme, CD39 can participate in ADO production through ATP metabolism [33], and its anti-inflammatory effect is related to maintaining a high level of extracellular adenosine (ADO). Activation of the transcription factor CREB will increase the expression of CD39 on circulating regulatory T cells (Tregs). Over-expression of EGR-1 in RA synovial fibroblasts may promote synovial fibrosis in RA by activating genes encoding α1 and α2 chains of type I collagen, and at the same time, it can regulate the production of proinflammatory factors through synergistic mechanisms and other pathways [34,35,36,37] (Figure 6B).

The compound 1-(9Z-octadecenoyl)-sn-glycerol-3-phosphocholine in the glycerophosphate metabolic pathway can be transformed into lecithin under the influence of lysophosphatidylcholine acyltransferase (LPCAT), and lecithin can be transformed into phosphatidylserine under the influence of phosphatidylserine synthase 1 (PTDSS1) (Figure 6C). Apoptosis can be roughly divided into four stages: apoptosis signal transduction, activation of apoptotic genes, execution of apoptosis, and elimination of apoptotic cells. Michael R. Elliott [38,39] vividly summarized it into four stages: looking, eating, devouring, and processing. Each stage is subject to strict signal control to ensure rapid and effective removal. Phosphatidylserine (PtdSer) is a key “eat me” signal. PtdSer will activate GTPase Rac, and the cell membrane will undergo morphological changes to devour dying cells. Finally, phagocytosis lysosomes process dying cells to degrade them. Liposomes that express PtdSer have been shown to mimic some immune-modulating responses to apoptotic cells [40]. Research indicates that soybean-derived phosphatidylserine reduces inflammation in RA-FLS cells activated by IL-1β in vitro [41].

## 4. Materials and Methods

### 4.1. Instruments and Reagents

Biosafety cabinet (HR90-IIA2, Haier, Qingdao, China); Carbon dioxide incubator (CCL-170b-8,ESCO, Jinan, China); Inverted microscope (CKX53, Olympus, Tokyo, Japan); Low-speed centrifuge (5424R, Eppendorf, Hamburg, Germany); Multifunctional enzyme label instrument (SynergyHTX, BIO-TEK, Winooski, VT, USA); 1000 μL, 200 μL, 100 μL, and 10 μL pipettes (Transferpette, Hamburg, Germany); Vacuum safety suction pump (DSP1000, SCILOGEX, Hartford, CT, USA); Pap pipette (23320767, Biosharp, Hefei, China); T25 cell culture flask (BIOFIL, Guangzhou, China); Fetal bovine serum (2287582CP, Gibco Ltd., GrandIsland, NE, USA); DMEM (6123070, Gibco Ltd., GrandIsland, NE, USA); Special culture medium for FLS cells (RT-003, Shanghai Jinyuan Biotechnology, Shanghai, China); PBS buffer (20230614, Nanjing KeyGen Biotech, Nanjing, China); Trypsin (2661767; Gibco Ltd., GrandIsland, NE, USA); TNF-α (Novoprotein Scientific Inc.,Suzhou, China); Dimethyl sulfoxide (20240412, Nanjing KeyGen Biotech, Nanjing, China); Berberine (DSTDX000901, Lemeitian medicine, Chengdu, China); CCK-8 reagent (20231211, Jiangsu KeyGEN BioTECH Corp. Ltd., Nanjing, China).

### 4.2. Cell Culture and Treatment

The cell source is the synovial tissue of human joints obtained by surgery, and the culture system is the primary fibroblast culture system. Cell batch number: 202309 was purchased from Shanghai Jinyuan Biotechnology. After receiving the RA-FLS cells, the bottle wall was sterilized with 75% alcohol, the T25 cell culture bottle was placed in an incubator at 37 °C for about 2–3 h, and the growth and adhesion of the cells were observed under a microscope. When the cell growth density reached more than 80%, FLS special culture medium and pancreatin were used to subculture the cells. Fluid changes were performed daily, and passages were made once every 2–3 days and inoculated into T25 cell culture flasks containing 10% fetal bovine serum in DMEM. Subsequent experiments were performed after three passages.

Preparation of berberine solution: A quantity of 5 mg of berberine standard powder was dissolve in 50 μL of DMSO; basal medium was added up to 5 mL to form a mother solution of 297.3 mM and was stored in the refrigerator at −20 °C. Berberine solution was diluted to the concentrations of 25 μM, 50 μM, and 100 μM using a basal medium containing 10 ng/mL TNF-α, which was used as a working solution.

### 4.3. Cytotoxicity Detection

The effect of berberine on FLS cell viability was detected by CCK-8 reagent. The FLS cells were adjusted to a cell concentration of 1 × 10^4^ cells/mL, inoculated into 96-well plates with 100 μL per well and four replicate wells per group, and incubated in a 5% CO_2_ incubator for 24 h at 37 °C. Berberine solution was added at concentrations of 0, 25, 50, and 100 μM, respectively, and incubated for 24 h. After that, 10 μL of CCK-8 reagent was added to each well and incubated for 60 min. Measurement of absorbance at 450 nm was performed using a multifunctional enzyme marker.

### 4.4. Flow Cytometry Detection

The FLS cells were subjected to treatment for 24 h, in accordance with the concentration administered. The cells were subjected to centrifugation at 300× *g* for 5 min. After collection, they were washed once with PBS. A quantity of 1–5 × 107 resuspended cells was taken and subjected to centrifugation at 300× *g* for 5 min. The cells were collected again and washed once with PBS. The supernatant was discarded, and the cells were resuspended by adding 100 μL of diluted 1× Annexin V Binding Buffer. A volume of 2.5 μL of Annexin V-FITC reagent and a volume of 2.5 μL of PI reagent (50 μg/mL) were combined with the cell suspension. The solution was gently mixed by vortexing to ensure thorough mixing. The mixture was incubated for 15–20 min at room temperature, away from light. A volume of 400 μL of diluted 1× Annexin V Binding Buffer was taken and thoroughly combined with the sample. Ultimately, the cells that were resuspended were collected by a machine and examined with Novo software.

### 4.5. Apoptosis Detection by Confocal Laser Scanning Microscope

Cells were cultured in laser confocal microscopy dishes to a density of 70–80%, and FLS cells were treated according to the administered concentration for 24 h. The original medium was discarded, and the cells were rinsed three times with pre-cooled PBS. Then, 200 μL of diluted 1× Annexin V Binding Buffer was added, along with 10 μL of Annexin V-FITC reagent and 10 μL of PI reagent. The cells were gently mixed and incubated for 15 min at room temperature, while being shielded from light. Next, 50 μL of diluted 1× Annexin V Binding Buffer was added. Finally, apoptotic cells were collected using an FV3000 laser confocal microscope (Olympus, Tokyo, Japan).

### 4.6. Sample Processing of FLS Cell Exosomes or Extracellular Lipomics Analysis

FLS cells were inoculated in 25 cm^2^ culture flasks and incubated in a 5% CO_2_ incubator at a temperature of 37 °C for a period of 24 h until the cell density achieved 70–80%. The original medium was discarded, and the cells in the model group were cultured using a basic medium containing 10 ng/mL TNF-α. The berberine group was administered at a concentration of 50 μM (prepared using a basic medium with 10 ng/mL TNF-α). After incubation for 24 h, the medium was discarded. Then, 1 mL of trypsin was given to digest the cells. The mixture is rotated at a speed of 3000 rpm for 5 min. Discard the supernatant. After washing twice and resuspending in PBS, the sample was quenched by liquid nitrogen. Then store it at 80 °C. Take samples from 80°C and thaw them on ice (all subsequent procedures must also be carried out on ice). Then, 500 μL of 80% methanol water internal standard extract was introduced and vigorously mixed for 3 min using a vortex. The samples were rapidly frozen in liquid ammonia for a duration of 5 min and then subsequently thawed on dry ice for 5 min. After that, they were totally thawed on ice and mixed vigorously for 2 min using a vortex. This process was performed three times. The centrifuge was spun at a speed of 12,000 r/min for a duration of 10 min at 4 °C. The entire liquid portion (supernatant) was transferred into another tube that had the same number and concentrate in it until the supernatant was completely devoid of moisture. The desiccated powder was dissolved again in 100 μL of a solution containing 70% methanol and water. The mixture was vigorously mixed for 3 min using a vortex mixer and then subjected to ultrasonic waves in an ice water bath for 10 min. The specimen underwent centrifugation at a speed of 12,000 r/min for a duration of 3 min at 4 °C. Subsequently, 80 μL of the resulting liquid above the sediment was transferred using a pipette into a tube that was equipped with the appropriate injection bottle for subsequent instrumental analysis.

### 4.7. UPLC-QTOF-MS Analysis

The cell samples underwent analysis using Sciex-ExionLC AD ultra-performance liquid chromatography and AB SCIEX QTRAP mass spectrometry. The chromatographic separation was performed using a Waters ACQUITY UPLC HSS T3 C18 column with dimensions of 1.8 µm and 2.1 mm × 100 mm The mobile phase was composed of ultrapure water (A) with 0.1% formic acid and ultrapure water of acetonitrile (B) with 0.1% formic acid. The flow rate was adjusted to 0.4 mL/min. The gradient settings for the phase of motion were as follows: from 0 to 11 min, the concentration of B increased from 5% to 90%; from 11 to 12 min, the concentration of B remained at 90%; from 12 to 12.1 min, the concentration of B decreased from 90% to 5%; and from 12.1 to 14 min, the concentration of B remained at 5%. The column temperature remained around 40 °C, while the injection rate was 2 μL.

The detection of mass spectrometry was performed in either positive or negative ion modes. The following settings were configured: the electrospray ion source temperature was set to 500 °C, and the mass spectrometry voltage was set to 5500 V for positive ions and −4500 V for negative ions. The ion source gas I and gas II were set to 50 psi each, while the curtain gas was set to 25 psi. Additionally, the collision-activated dissociation parameter was set to high. Within the QTRAP system, every ion pair undergoes scanning and detection based on carefully optimized declustering current and collision energy settings.

### 4.8. Metabolite Identification

The chromatographic separation and mass spectrometric analysis were performed by Wuhan Metavir Biotechnology Co. Ltd. (Wuhan, China). using a data acquisition instrument system. Based on the self-constructed specimen database MWDB (including secondary spectra and retention time RT), the DB-all public database (including Metlin, HMDB, KEGG, and other databases), the AI prediction library, and the MetDNA database were integrated to carry out accurate characterization and to extract information such as multiple ion pairs and retention time RT from the identified metabolites and were then combined with Meinville’s self-constructed target database in order to form a new library exclusive to the project. Finally, in all the samples, the metabolites in the library were accurately quantified by MRM based on the QTRAP instrument platform.

### 4.9. Pattern Recognition Analysis

Using the “PCA and OPLS-DA” module in the cloud tools of the OmicShare, the peak detection and peak alignment data were normalized to obtain PCA score plots of exosomal samples reflecting the degree of inter-group dispersion in order to detect intra-group commonalities and inter-group disparities. On the basis of PLS-DA, orthogonal transformations are corrected to filter out the noise unrelated to the classification information to obtain the OPLS-DA score map. Without reducing the model’s predictive power, OmicShare successfully minimizes the complexity of the model while increasing its explanatory ability, allowing for a clearer perspective of the variances between groups.

### 4.10. Data Visualization Analysis

The component name, mass spectrum peak intensity information, lipid classification information, and group information of the core difference metabolites between the model group and the berberine group are first imported into the “normalization preprocessing” module in the cloud tool of BioDeepto preprocess the data, including missing value filtering, missing value filling, and data normalization, and then imported into the “cluster heat map” module and the “correlation heat map” module for analysis. At the same time, the data are imported into the data processing platform of MetaboAnalyst 5.0, and the module of DSPC is adopted. The module algorithm analyzes the correlation of core biomarkers based on the modeling process of sparse graphics lasso. The system is a combination of gene, metabolite, and disease interaction systems. In addition, the Enrichment Analysis module in MetaboAnalyst, a data processing platform, is used to enrich and analyze the core biomarkers.

### 4.11. Data and Statistical Analysis

The data were presented as means ± SEM. Multiple comparisons were made using one-way ANOVA and Dunnett’s multiple comparisons test. A *t*-test was employed for contrasting the two groups. *p*-values < 0.05 have been deemed to be significantly different. The statistical results were analyzed using the GraphPad Prism 10 software.

## 5. Conclusions

Based on the beneficial effects of berberine for treating RA, the lipid metabonomics method of RA-FLS exosomes was established for the first time, and the core biomarkers in exosomes closely related to RA were analyzed. It was found that berberine could regulate 11 biomarkers, including LPE (16:0/0:0), LPE (0:0/20:4), LPE (20:4/0:0), LPE (0:0/16:0), and LPE (22.6/0:0), 1-(9Z-octadecenoyl)-sn-glycero-3-phosphocholine, 12-methyltridecanoic acid, FFA(16:0), 16-hydroxyhexadecanoic acid, 2-hydroxyhexadecanoic acid, and 1-dodecanoyl-glycero-3-phosphate. Eleven biomarkers were projected into multiple visual databases based on multi-source integration and interaction, and it was found that berberine may affect lipid metabolism pathways such as glycerophosphate metabolism, the biosynthesis of unsaturated fatty acids, and fatty acid extension, degradation, and biosynthesis. Finally, it was found that berberine may inhibit the proliferation and inflammatory reaction of RA-FLS cells by affecting the binding rate of the PPARγ-NF-κB complex, the expression of CREB and EGR-1, the biogenesis and maintenance of organelles, and cell phagocytosis, thus exerting its therapeutic effect on RA. The above results show that berberine, a new therapeutic drug targeting exosomes or drug delivery systems, is expected to become an effective drug for RA.

## Figures and Tables

**Table 1:** Lipid biomarkers and change trends in extracellular matrix of model group and berberine group.

NO.	*m*/*z*	Adducts	Formula	Compound	LIPIDMAPS ID	Trend
1	452.3000	C_21_H_44_NO_7_P	[M–H]^−^	LPE(16:0/0:0)	LMGP02050002	↑ *
2	246.2433	C_14_H_28_O_2_	[M+NH_4_]^+^	12-Methyltridecanoic acid	LMFA01020007	↓ *
3	255.2000	C_16_H_32_O_2_	[M–H]^−^	FFA(16:0)	LMFA01010001	↓ *
4	500.3000	C_25_H_44_NO_7_P	[M–H]^−^	LPE(20:4/0:0)	LMGP02050009	↑ **
5	452.3000	C_21_H_44_NO_7_P	[M–H]^−^	LPE(0:0/16:0)	LMGP21050007	↑ **
6	500.3000	C_25_H_44_NO_7_P	[M–H]^−^	LPE(0:0/20:4)	LMGP02050051	↑ **
7	271.2000	C_16_H_32_O_3_	[M–H]^−^	16-Hydroxyhexadecanoic acid	LMFA01050051	↓ **
8	271.2000	C_16_H_32_O_3_	[M–H]^−^	2-Hydroxyhexadecanoic acid	LMFA01050047	↓ **
9	524.3000	C_27_H_44_NO_7_P	[M–H]^−^	LPE(22:6/0:0)	LMGP02050013	↑ **
10	580.3569	C_26_H_52_NO_7_P	[M+CH_3_COO]^−^	1-(9Z-octadecenoyl)-sn-glycero-3-phosphocholine	LMGP01050032	↑ **
11	431.0955	C_15_H_31_O_7_P	[M–H+2K^]+^	1-Dodecanoyl-glycero-3-phosphate	LMGP10050015	↓ **

Note: “↑” means that the differential metabolites in the berberine group are on the rise compared with the model group, and “↓” means that the differential metabolites in the berberine group are on the decline compared with the model group. Compared with the model group, * *p* < 0.05, ** *p* < 0.01.

## Data Availability

The original contributions presented in the study are included in the article, further inquiries can be directed to the corresponding authors.
